# PyPop: a mature open-source software pipeline for population genomics

**DOI:** 10.3389/fimmu.2024.1378512

**Published:** 2024-04-02

**Authors:** Alexander K. Lancaster, Richard M. Single, Steven J. Mack, Vanessa Sochat, Michael P. Mariani, Gordon D. Webster

**Affiliations:** ^1^Amber Biology LLC, Cambridge, MA, United States; ^2^Ronin Institute, Montclair, NJ, United States; ^3^Institute for Globally Distributed Open Research and Education (IGDORE), Cambridge, MA, United States; ^4^Department of Mathematics and Statistics, University of Vermont, Burlington, VT, United States; ^5^Department of Pediatrics, University of California, San Francisco, Oakland, CA, United States; ^6^Livermore Computing, Lawrence Livermore National Laboratory, Livermore, CA, United States; ^7^Mariani Systems LLC, Hanover, NH, United States

**Keywords:** HLA, MHC, population genomics, software, bioinformatics

## Abstract

Python for Population Genomics (PyPop) is a software package that processes genotype and allele data and performs large-scale population genetic analyses on highly polymorphic multi-locus genotype data. In particular, PyPop tests data conformity to Hardy-Weinberg equilibrium expectations, performs Ewens-Watterson tests for selection, estimates haplotype frequencies, measures linkage disequilibrium, and tests significance. Standardized means of performing these tests is key for contemporary studies of evolutionary biology and population genetics, and these tests are central to genetic studies of disease association as well. Here, we present PyPop 1.0.0, a new major release of the package, which implements new features using the more robust infrastructure of GitHub, and is distributed via the industry-standard Python Package Index. New features include implementation of the asymmetric linkage disequilibrium measures and, of particular interest to the immunogenetics research communities, support for modern nomenclature, including colon-delimited allele names, and improvements to meta-analysis features for aggregating outputs for multiple populations.

Code available at: https://zenodo.org/records/10080668 and https://github.com/alexlancaster/pypop

## 1 Introduction

Since its principles were established a century ago (1–5), population genetics has been a computational science. The advent of electronic computing, and its widespread adoption for academic research in the 1980s and 1990s, fostered the development of computational genetics software (e.g., 6, 7) that could perform multiple analyses and return results in standardized, human and machine-readable formats. PyPop (Python for Population Genomics) was initially developed between 2002 and 2007 (8, 9) as a Python 2-based framework that performed multiple population genetic analyses on highly-polymorphic, multilocus genotype data, and generated both standardized, “publication ready” text-formatted outputs and machine-readable XML outputs, allowing for further downstream analyses and meta-analyses.

A standard PyPop analysis is initiated by running the “pypop” command-line program that is supplied with one or more plaintext input “population” or “dataset” files (with the suffix “.pop”), along with a plaintext input configuration file (with the suffix “.ini”). The input configuration file defines both the expected input format, as well as the specific analyses that will be run, including tests of Hardy-Weinberg equilibrium expectations, Ewens-Watterson tests of selection, and estimation of haplotype frequencies and linkage disequilibrium [a full list of the configuration options is available in the *PyPop User Guide* (10)]. Each input file results in a corresponding set of output files: a machine-readable XML file, and a human readable plain-text file. These primary analyses can be aggregated to generate cross-dataset meta analyses using “popmeta”, another tool in the PyPop suite. Here, we describe PyPop version 1.0.0, which is built using Python 3 and includes new features and improvements as well as a new development platform.

We first document the ongoing use of PyPop in the immunogenetics and other research communities in the years since the last release of PyPop (version 0.7.0). Next we describe new features and analytical methods, including measure of asymmetric linkage disequilibrium (ALD), and updates to support the current nomenclatures for major histocompatibility complex (MHC) and human leukocyte antigen (HLA) genes. We also note the streamlining and improvement of existing features such as the custom grouping of alleles and output of tab-separated value (TSV) files. We close by describing features in development, as well the porting of the project to GitHub to support future Python versions and new machine architectures, providing a stable home for PyPop to evolve as a community resource.

## 2 Methods and results

### 2.1 PyPop in the human immunogenetics community and beyond

**Figure 1:**
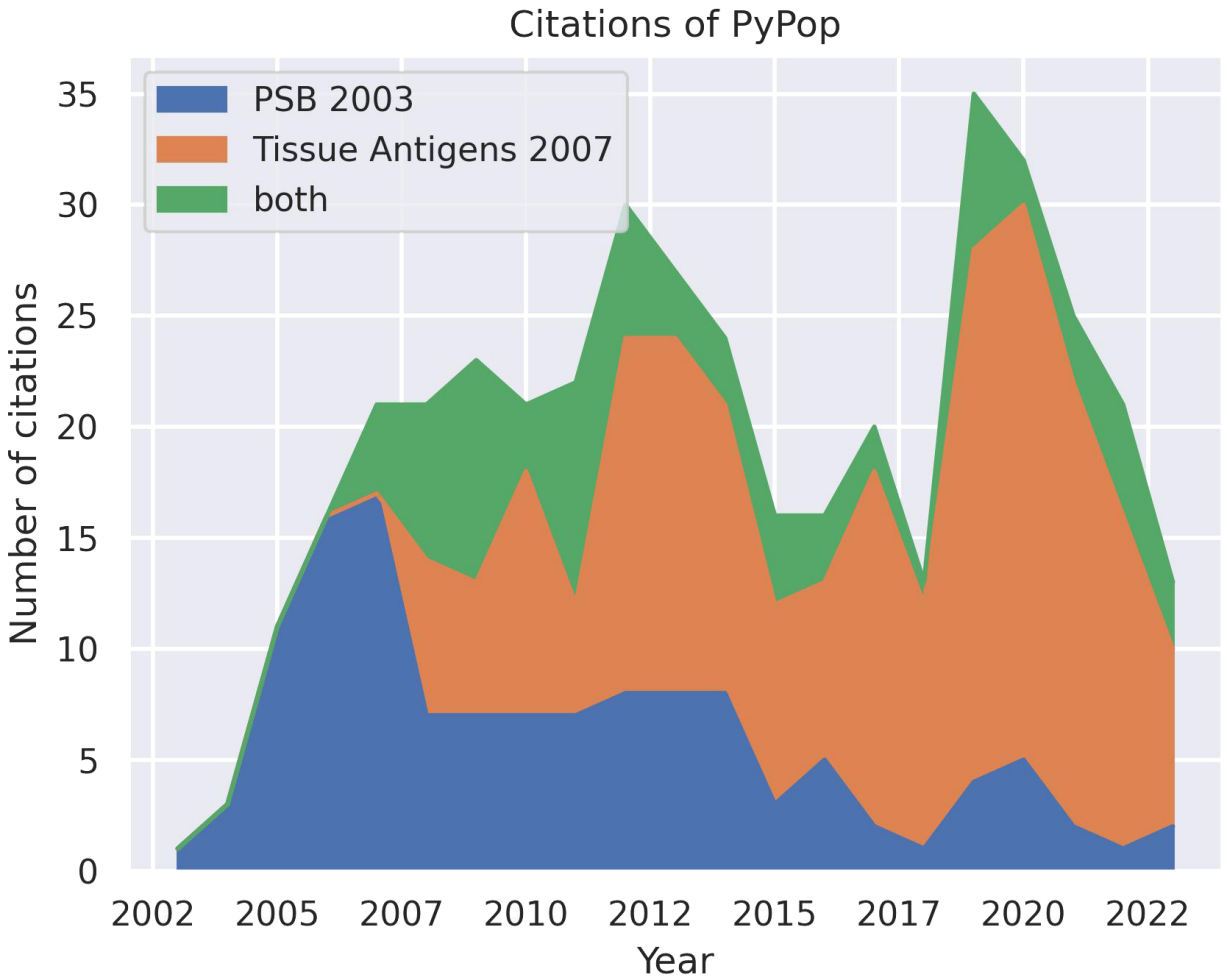
Number of unique citations over time for the two previous PyPop publications: *Pacific Symposium in Biocomputing* (*PSB*) (8) and *Tissue Antigens* (9). Some publications cited both PyPop papers. Google Scholar was used for the counts.

Since the first public release of the software in 2003 and the subsequent publication of descriptions in 2003 (8) and 2007 (9), PyPop has been in regular and continuous use within the HLA and the larger genomics communities, as shown in an analysis of Google Scholar citations (**Figure 1**). This analysis estimates that there have been 433 unique citations of PyPop since its inception (134 for the 2003 paper alone, 220 for the 2007 paper, and 79 for both). Of those unique citations, 367 are from 2007 or later. PyPop has been applied extensively within the immunogenetics community since its first release, as expected given its origins as part of the 13th International Histocompatibility Workshop (IHWS) in 2002 (11). A notable early meta-analysis of the action of natural selection on HLA polymorphism across 497 populations (12), relied heavily on PyPop 0.7.0 analyses and has 360 citations in Google Scholar at the time of writing.

Many of these citations are from researchers studying human immune system genes. However, PyPop has been used in many studies, far from its home research community. These include studies that are both taxonomically distinct (genetic heterogeneity of urban foxes (13)) and genetically distinct [population genetics of cytochrome enzyme proteins (14)] from human immunogenetics. These two examples illustrate the wide utility of PyPop as a computational population genomics resource.

### 2.2 New features and improvements

#### 2.2.1 Asymmetric linkage disequilibrium measures

The conditional asymmetric linkage disequilibrium (ALD) measures, first described by Thomson and Single (15), are the major new analytic feature of PyPop 1.0.0. Previous PyPop versions computed two measures of overall linkage disequilibrium: *D’* (16), which uses the product of pairwise allele frequencies to weight the individual haplotype-level coefficients of LD, and *W_n_* (17), which is a multi-allelic extension of the “*r”* correlation measure commonly used for LD with bi-allelic SNPs. ALD further extends the *W_n_* measure, accounting for asymmetries that arise from different numbers of alleles at different loci. The two measures, *W_12_* and *W_21_*, assess LD conditional on the second and first locus, respectively, and are both equal to the usual *r* statistic for SNPs (**Table 1**).

**Table 1:** Comparison of the default text-based output for a single two-locus pairwise LD measures for a pre-1.0.0 version (a) and 1.0.0 version (b) of PyPop, which include the new ALD measures, *W_12_* and *W_21_*, denoted by ALD_1_2 and ALD_2_1 in the output, respectively. 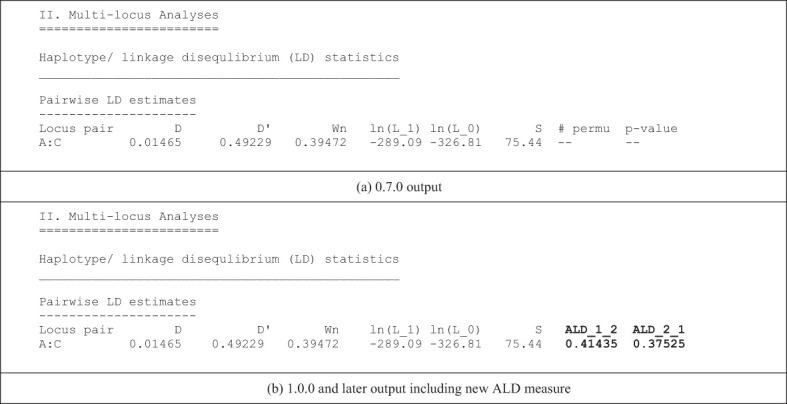

Note that the # permu and p-value columns are now only displayed if a permutation test is run.

ALD is particularly useful when investigating LD in highly polymorphic gene-systems, where each locus displays large and very different numbers of alleles in a population. These ALD measures, computed using PyPop, have been used in anthropological studies dissecting LD in human populations (18, 19); studies of permissible mismatches in HLA donor registries (20); and studies of HLA haplotypes and amino acid motifs that predispose for disease (21). Additional publications, using different implementations of the ALD, include studies of the impact of anti-malarial drugs on parasite populations among individuals with complex infection status (22, 23). ALD measures allow one to condition on known disease genes in association studies when searching for additional genetic effects in a region. Similarly, by conditioning on putative targets of selection ALD measures can help characterize other potentially selected variants.

#### 2.2.2 Support for modern HLA/MHC nomenclature

Since the major release of PyPop 0.7.0 in 2008, the allele-name nomenclatures for MHC and HLA genes have changed significantly. In 2010 (24) the format of HLA and MHC allele names was changed to include colon-delimited fields, where previous formats had relied on ‘digit-based’ fields. An allele denoted as 0101 before 2010 is now denoted as 01:01. This nomenclature change also means that much longer HLA allele names (eg., A*02:01:01:134Q or DPB1*1372:01:01:02) are now valid, and PyPop can continue to process such data. In addition, the ~ operator, defined in the text-based Genotype List (GL) String syntax for describing HLA and Killer-cell Immunoglobulin-like receptor (KIR) genotyping results (25, 26), has been the standard for delimiting alleles in multi-locus haplotypes with the immunogenetics community. In PyPop 1.0.0, a two locus haplotype of alleles at two loci, A and B respectively, is represented as A~B, where this haplotype had been represented as A:B in earlier PyPop releases.

Although previously there was nothing actively preventing a user of PyPop from using the 2010 HLA/MHC nomenclature for PyPop input data, PyPop 0.7.0’s separation of haplotype elements with colons meant that a “:” *within* an allele name could lead to ambiguous output. We introduced changes in version 1.0.0 to seamlessly handle the 2010 nomenclature, and now PyPop output includes the GL String ‘~’ separator by default, facilitating use of the output in publications or further downstream analyses (**Table 2**). We have updated all documentation, examples and unit tests to reflect these changes.

**Table 2:** Comparison of haplotype estimation output indicating use of both the new nomenclature and the GL String haplotype separator. 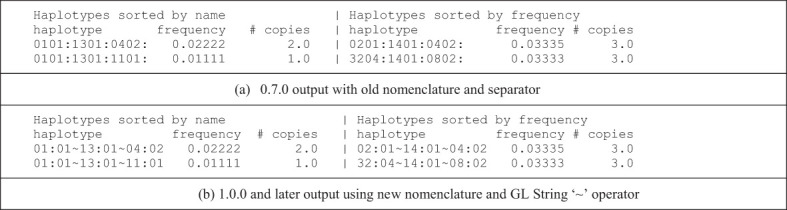

#### 2.2.3 Cross-platform support for custom grouping (“binning”) filters

PyPop’s capacity for “custom binning”, which combines allele-names into specific categories for analysis, is now available on all platforms. This capacity extends to commonly used allele groupings (e.g., G- and P-groups (24), supertype groups (27), HLA T-cell epitope (TCE) groups (28, 29), and National Marrow Donor Program [NMDP] allele codes (30, 31)) that group distinct variants by common aspects. For example, as of January 2024, the A*01:01:01G G-group designation represents 240 HLA-A alleles that share identical exon 2 and exon 3 nucleotide sequences. Supertypes are groups of alleles with similar peptide-binding features; for example DPB1 alleles with identical peptide sequences for amino-acid positions 11, 69 and 84 are sorted into eight supertypes groups (27).

TCE groups identify sets of DPB1 alleles with shared amino acid motifs that result in permissive mismatches in the context of hematopoietic stem cell transplantation (29). NMDP allele codes identify groups of alleles that cannot be distinguished by genotyping methods that do not sequence the entire HLA gene. For example, the DRB1*11AD allele code is used to represent a genotyping result that could be either DRB1*1101 or DRB1*1104 (31).

**Figure 2:**
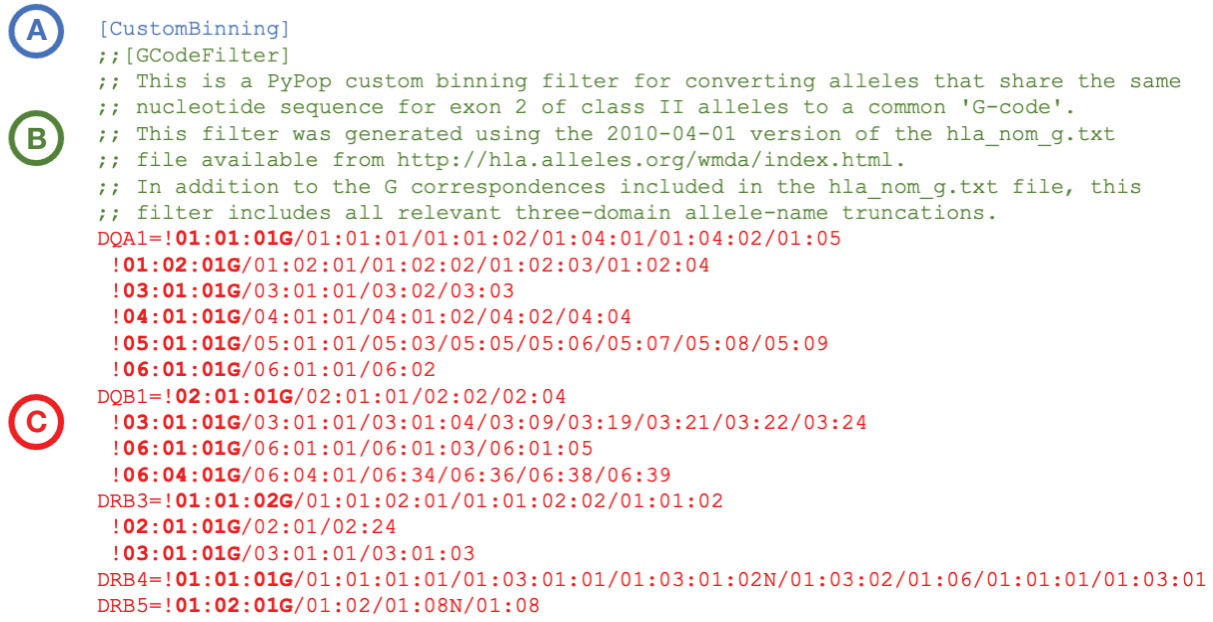
Extract of an example PyPop “CustomBinning” filter that could be included within the configuration “.ini” file for a PyPop run. The three elements of a custom binning filter for five HLA loci are shown. **(A)**: Header block. Every custom binning filter begins with the [CustomBinning] keyword. **(B)**: Comment block (optional). Comments are indicated with double semicolons. This comment block identifies the type of filter (here, “GCode”) and includes specific details about the source of the data used to inform the filter. **(C)**: Filters block. Filters for DQA1, DQB1, DRB3, DRB4 and DRB5 are shown. Each filter starts with an exclamation point, which is followed by the group identifier (shown in bold). The group identifier and its constituent alleles are delimited by forward slashes. Multiple groups for a locus are defined on separate lines, and all groups after the first start with a whitespace. When the filter is applied, any alleles in the dataset that are in a group will be converted to the group identifier for PyPop analysis. (A full plain-text version with additional rules for more loci - including the five shown - is contained in the file “G-Filter_config.ini” within **Supplementary File 1**).

PyPop custom binning is not restricted to these specific community-defined examples; variant names can be combined into any user-defined category for PyPop analysis. An example custom binning filter for converting alleles to a G-group designation is presented in **Figure 2**. Additional examples are provided in **Supplementary File 1**.

#### 2.2.4 Improved support for downstream analyses: enhancements to TSV output

PyPop analyses are always output as machine-readable XML files, with one XML file per population or dataset. Previous versions of PyPop included a feature to aggregate these individual dataset or population-level XML files into a set of files in tab-separated value (TSV) format, suitable for input into spreadsheets or other downstream software (**Table 3**). However, this feature was originally tuned to the needs of the 13th IHWS (11) and required adaptation for use outside this context. In PyPop 1.0.0, we have overhauled and re-tooled the output mechanism for general use. The changes include:

1. Previously the list of output TSV files was hardcoded, and this set of files was generated regardless of whether the analysis created any relevant data. For example, a 3-locus-haplo.tsv file was generated even if estimation of 3 locus haplotypes was not requested by the user - resulting in a file with headers, but no data. The output files are now dynamically generated based on the analyses that were requested by the user (ultimately based on aggregating the contents of the separate XML outputs generated by each input.pop dataset). In addition, we have also enabled generation of TSV output for haplotype estimation involving five or more loci, e.g. 5-locus-haplo.tsv, 6-locus-haplo.tsv, etc. (see the last two rows of **Table 3**).
2. Output files now use the standard “.tsv” suffix (rather than “.dat”) so they are more easily identified as tab separated value files that are parsable by other software. We have also renamed the command-line options accordingly (e.g. –generate-dat to –enable-tsv).
3. Previous versions included fixed metadata columns that were only relevant for the analyses performed for the 13th IHWS. These additional columns are now disabled by default (we have added a new “–enable-ihwg” option which will re-enable them).
4. We have added new options to enable TSV files to be saved in a separate directory (–outputdir) and include a prefix (–prefix-tsv).

**Table 3:** List of possible types of TSV files, their row data type and a brief description, including the generation of files containing multi-locus analyses with an arbitrary number of *n* loci.

Default file name suffix	Row data	Description
1-locus-summary.tsv	locus	Consists of a line for population and locus, with fields for number of gametes, number of distinct alleles, HWP p-value for the Chi-square test and all other single locus statistics.
1-locus-allele.tsv	allele	Consists of a line for each combination of population, locus and allele. The line of data contains the allele frequency (allele.freq) and count (allele.count)
1-locus-genotype.tsv	genotype	Consists of a line for each combination of population, locus and genotype, with individual genotypes statistics (only output if individual statistics are selected by the user)
1-locus-hardyweinberg.tsv	locus	Consists of a line for each population and locus, with fields for number of distinct alleles and several versions of computing p-values for HWP (Guo and Thompson original and monte-carlo method, full enumeration when possible, heterozygotes, homozygotes)
2-locus-summary.tsv	locus	Consists of a line for each combination of population, and locus group. Columns representing locus-level statistics. If a pairwise analysis has been requested, it will also include the pairwise LD statistics discussed above, D’, W_n_ and ALD_12_, ALD_21_.
2-locus-haplo.tsv	haplotype	This is analogous to the 1-locus-allele.tsv, except with information for each population’s haplotype, such as the estimated haplotype count and frequency. If pairwise analysis has been selected, it will also include individual haplotype D’ and W_n_ measures.
*n*-locus-summary.tsv	locus	Analogous to the 2-locus-summary.tsv output, but no pairwise statistics
*n*-locus-haplo.tsv	haplotype	Analogous to the 2-locus-haplo.tsv output, but omits the individual pairwise LD measurements

These changes should increase the utility of PyPop for meta-analyses in a wider range of research use-cases, particularly for studies that need to aggregate analyses where haplotypes were estimated at more than four loci.

### 2.3 Development updates

When PyPop development started in late 2001 Python was at version 2. Soon after the last release of PyPop (0.7.0) in 2008, Python 3 was released. Python 3 unfortunately introduced breaking changes (breaking the existing PyPop code). With the end-of-life of Python 2 in 2020, migration from PyPop to Python 3 became an imperative. In addition to the new scientific features described above, and the desired transition to Python 3, other major goals of the PyPop 1.0.0 release were (a) to improve ease of installation and the overall experience for end-users, (b) to make it easier to contribute to PyPop, and (c) reduce “technical debt” (32) and thus improve overall project longevity. In this section, we discuss these changes to the development process, the Python 3 migration, improvements in packaging, deployment, provenance, and documentation to further these end-goals.

#### 2.3.1 Development moved to the GitHub platform

In 2013 we migrated the source code version control system of PyPop from an internal Concurrent Versions System (CVS) repository to Git, and subsequently imported it as a public project on the GitHub platform. GitHub supports advanced features for developers including issue and milestone tracking, discussions, collaborative code review (pull requests), security scanning, and automation of testing via continuous integration (CI). With this change, the development process became more open to the community. Updates that added support for codon-delimited alleles and increased capacity for multi-locus analyses were made as part of the 17th International HLA & Immunogenetics Workshop, which was held in 2017 (33) and made available via GitHub, although no formal release was made at this time.

#### 2.3.2 Migration to Python 3

Migration commenced in 2017, by an author of this paper (34) - outside the original development team - via a “pull-request”, illustrating the benefits of moving to the GitHub platform. Initially the process was largely manual, including fixing of print statements, addition of modules, and rearranging of module imports. We included Singularity (35), an upcoming container technology for high performance computing, and a pull request to update from the deprecated “Numeric” to the “numpy” library was merged later in 2017 (36). In early 2023, we merged a modified version of the pull request, including additional changes, back into the main branch, which finalized the conversion to Python 3.

#### 2.3.3 New test suite and continuous integration

During the port, we created a test suite that included both unit tests, and end-to-end “pipeline” tests, emulating end-user runs. As a result of this process, we refactored code, and removed obsolete or outdated code, helping to reduce technical debt. Apart from its direct utility in detecting regressions introduced during development, this test suite has resulted in a wider set of configuration (“.ini”) and data (“.pop”) files that provide examples for end-users of PyPop to emulate. We also leveraged GitHub’s CI feature, known as GitHub Actions, so that these tests are automatically run upon a commit to the repository.

#### 2.3.4 Generating source distributions and binary wheels for Windows, MacOS X and Linux

The cibuildwheel system (37) generates “wheels” (architecture-specific installable versions of a Python package containing pre-compiled extensions), installs each wheel in a virtual environment, and then runs unit tests within the virtual environment with that installed wheel. Key to this process is that cibuildwheel automates the process of compiling and testing wheels across multiple operating systems and Python versions, ensuring that they will work on each of those end-user systems. We deployed cibuildwheel as part of our GitHub Action workflow, resulting in over 40 different tested wheels on a wider range of architectures and Python versions (**Supplementary Table 1**) - compared with only two binary packages available previously (one for Linux, and one for Windows). These wheels include, for the first time, an official pre-compiled MacOS X version of PyPop, on both Intel (x86) and Apple Silicon (arm64) architectures. In addition to the automated CI testing, we did manual testing on several Windows, Linux and Android platforms (**Supplementary Table 2**).

#### 2.3.5 Deploying releases via the Python Package Index (PyPI)

When a release is made via GitHub’s “tag-and-release” interface, our workflow triggers a build of all binary wheels and source distribution via GitHub’s CI system, as described above, but includes an additional step in the workflow of uploading a versioned release to the PyPI repository. This vastly simplifies installation for end users who can install PyPop directly from PyPI via a single “pip install pypop-genomics” command.

#### 2.3.6 Provenance via Zenodo DOI

We configured the workflow so that, upon a production release via GitHub, it will deposit the source and metadata about the release to the Zenodo repository (38). This generates version-specific archives of the source code, together with a unique Digital Object Identifier [DOI]. Users can then cite the specific version used for their analyses as a DOI in their paper to enable more effective reproducibility (39). For example, the DOI for the 1.0.0 release being described in this paper is 10.5281/zenodo.1008066 (40).

#### 2.3.7 Maintainable documentation

The previous version of the *PyPop User Guide* (10) was written using DocBook XML (41) which, while powerful, has a steep learning curve. For this new release, we converted all documentation to reStructuredText (42) which, as a simple plaintext-like language, is more intuitive for contributors. We created another GitHub Action workflow that runs the sphinx documentation generator (43) to generate both HTML and PDF versions of the *User Guide* and the website from the reStructuredText documents. This GitHub workflow ensures that all changes are automatically deployed to the pypop.org website with each commit to the repository. In addition, some of the documentation (e.g. command-line options) is either generated directly from the code, or pulled in from configuration and data files in the unit tests, further ensuring that documentation is always kept in sync with the current codebase.

## 3 Discussion

PyPop development continues beyond this 1.0.0 release. A set of features in development related to the estimation of haplotype frequencies and LD include a reworking of the existing implementation of the Expectation-Maximization algorithm; computing LD between loci when allelic phase is known; and moving less computationally-intensive aspects of code currently implemented in C extensions into Python. This will allow for an increase in the number of loci for which haplotypes can be estimated, relative to the existing implementation, because the new implementation doesn’t require retention of all possible haplotype combinations. A preliminary, but incomplete implementation is already contained within PyPop 1.0.0 for alpha testing, but should not be used for production analyses.

Since the last release 16 years ago, PyPop has been in active and continuous use across a range of research communities. Despite a relative stasis in development during that period, PyPop has illustrated its durability as a framework for producing standardized reports for population genomics analyses. With the updated development platform, unit testing, packaging and deployment system in place, we have set a foundation to allow for more frequent, and well-tested releases, in addition to improving maintainability and encouraging contributions.

## Software information

- **Project links**: http://pypop.org (home page), https://github.com/alexlancaster/pypop/ (development page)
- **Operating systems**: Linux, MacOS X, Android, Windows
- **Programming languages**: Python and C
- **License**: GNU GPLv2: https://www.gnu.org/licenses/gpl
- Any restrictions for non-academic use? None
- **Zenodo record**: https://zenodo.org/records/10080668

## Data availability statement

Publicly available datasets were analyzed in this study. This data can be found here: https://zenodo.org/records/10080668 (Zenodo software archive).

## Author contributions

AL: Conceptualization, Formal analysis, Project administration, Software, Writing – original draft, Writing – review & editing, Investigation, Methodology, Validation, Visualization. RS: Conceptualization, Funding acquisition, Resources, Software, Writing – original draft, Writing – review & editing, Investigation, Project administration, Validation, Methodology. SM: Funding acquisition, Methodology, Writing – original draft, Writing – review & editing, Validation. VS: Software, Writing – review & editing, Writing – original draft. MM: Software, Writing – review & editing, Validation. GW: Software, Writing – review & editing.
