# Supplementary material for: PyPop: a mature open-source software pipeline for population genomics
Source: Front Immunol. 2024 Apr 2;15:1378512. doi: 10.3389/fimmu.2024.1378512 (PMC11019567; doi:10.3389/fimmu.2024.1378512)
Supplement: Supplementary file 1 [file DataSheet_1.pdf]

Supplementary Material

PyPop: A mature open-source software pipeline for population genomics

1 Supplementary Tables and Files

1.1 Supplementary Tables

Supplementary Table 1. Matrix of operating systems, architectures and Python versions that have binary packages ("wheels") available for PyPop at the time of writing, via `cibuildwheel`. "CPython" is the standard implementation of Python, "PyPy" (https://www.pypy.org/) is an alternative implementation of Python that claims to be faster than CPython. "manylinux" and "musllinux" are alternative implementations of the C library that are provided on different Linux distributions. End-users do not need to know which wheel to install, the "`pip install`" command will automatically select the wheel appropriate for the user's platform. Missing wheels (--) are generally a result of binary Python dependencies (mostly "Numpy") that are not available on that particular platform. If a user does wish to install on that platform, "`pip install`" will download the source tarball, but in order for this to succeed, it requires a full development environment, such as a compiler and other binary dependencies, to be installed. For this reason, we don't officially support that configuration. N/A means that the configuration is not available through `cibuildwheel`. Note that PyPop does not support any 32-bit architectures.

(Table appears on next page)

Supplementary Material

Operating system	macOS		Windows	Linux			
**Platform (architecture)**	**macOS Intel (x86_64)**	**macOS Apple Silicon (arm64)**	**Windows 64bit (amd64)**	**manylinux (x86_64)**	**manylinux (aarch64)**	**musllinux (x86_64)**	**musllinux (aarch64)**
CPython 3.6	✅	N/A	✅	✅	✅	--	--
CPython 3.7	✅	N/A	✅	✅	✅	--	--
CPython 3.8	✅	✅	✅	✅	✅	--	--
CPython 3.9	✅	✅	✅	✅	✅	✅	✅
CPython 3.10	✅	✅	✅	✅	✅	✅	✅
CPython 3.11	✅	✅	✅	✅	✅	✅	✅
CPython 3.12	✅	✅	✅	✅	✅	✅	✅
PyPy 3.7 v7.3	--	N/A	--	--	--	N/A	N/A
PyPy 3.8 v7.3	✅	--	✅	✅	✅	N/A	N/A
PyPy 3.9 v7.3	✅	--	✅	✅	✅	N/A	N/A
PyPy 3.10 v7.3	--	--	--	--	--	N/A	N/A

Supplementary Table 2. Platform specifications for manual testing of source builds, with full compilation, building and testing. Note that although there are wheels available for Windows and Linux, cibuildwheel does not yet provide Android-compatible wheels, and so PyPop installation on Android requires compilation from source.

<table>
<tr><th>Windows</th><th>Linux</th><th>Android</th></tr>
<tr><td rowspan="3">Machine: HP Z2 Workstation: Intel Core I5 8500 5th gen CPU @ 3.00 Ghz
8GB RAM, 500 GB hard drive
OS: Windows 11 Pro version 23H2 (64bit)
Python: 3.12.1</td><td>Machine: Virtual Box 7.0.14 (On Z2 workstation) 4GB RAM, 128 GB HD</td><td rowspan="3">Machine: Samsung Galaxy S22: Qualcomm Snapdragon 8 Gen 1 CPU @ 3Ghz, 8GB RAM, 128 GB hard drive
OS: Android 14, One UI: 6.0, Termux: 0.101
Python: 3.11.4</td></tr>
<tr><td>OS: Centos Stream 9 x86_64
Python: 3.9.18</td></tr>
<tr><td>OS: Ubuntu 22.04.3 LTS
Python: 3.10.6</td></tr>
</table>

1.2 Supplementary Files

Supplementary File 1. A zip file that contains six text files: **(A)** **`README`** with a detailed description of each file; **(B)** **`G-Filter_config.ini`**, **(C)** **`P-Filter_config.ini`**, minimal PyPop configuration files; **(D)** **`BIGDAWG_SynthControl_Data.pop`**, a synthetic input .pop file; **(E)** **`many_options_config.ini`**, a "maximal" ini file; and **(F)** **`BIGDAWG_SynthControl_Data_with_metadata.pop`**, another input .pop file. The most recent version of these files can be found on GitHub at: https://github.com/alexlancaster/pypop/tree/main/tests/data/custom-binning-examples and https://github.com/alexlancaster/pypop/tree/main/tests/data or in the current Zenodo archive: DOI: 10.5281/zenodo.10080667.
